# Four Independences

**Published:** 1871-04

**Authors:** 


### FOUR INDEPENDENCES.

To do without liquor.

To do without tobacco.

To do without warm water in shaving.

To do without going in debt.

Happy and great is the man who has force of will enough to put his foot on the ground and say, "It shall be done."

A pig would not get drunk twice; he who goes in debt sells his independence and his manhood, and is only fit to be under a despot's rule. As to the use of tobacco, it is so filthy, so disgusting, so beastly, that it is really a wonder that any gentleman could condescend to employ it either in chewing, snuffing, or smoking; and yet there are good men who are content to live under these slaveries. It has been said that a man may rid himself of the vice of chewing, by taking, after each meal, coarsely ground gentian root, chew it well, and swallow the saliva, for a few weeks.

